# Supplementary material for: Effectiveness and Safety of Ayurvedic Medicines in Type 2 Diabetes Mellitus Management: A Systematic Review and Meta-Analysis
Source: Front Pharmacol. 2022 Jun 8;13:821810. doi: 10.3389/fphar.2022.821810 (PMC9213670; doi:10.3389/fphar.2022.821810)
Supplement: Supplementary file 1 [file DataSheet1.zip › Supplementary Material/Forest and Funnel Plots/Trigonella foenum-graecum (versus OAD)/TG.pdf]

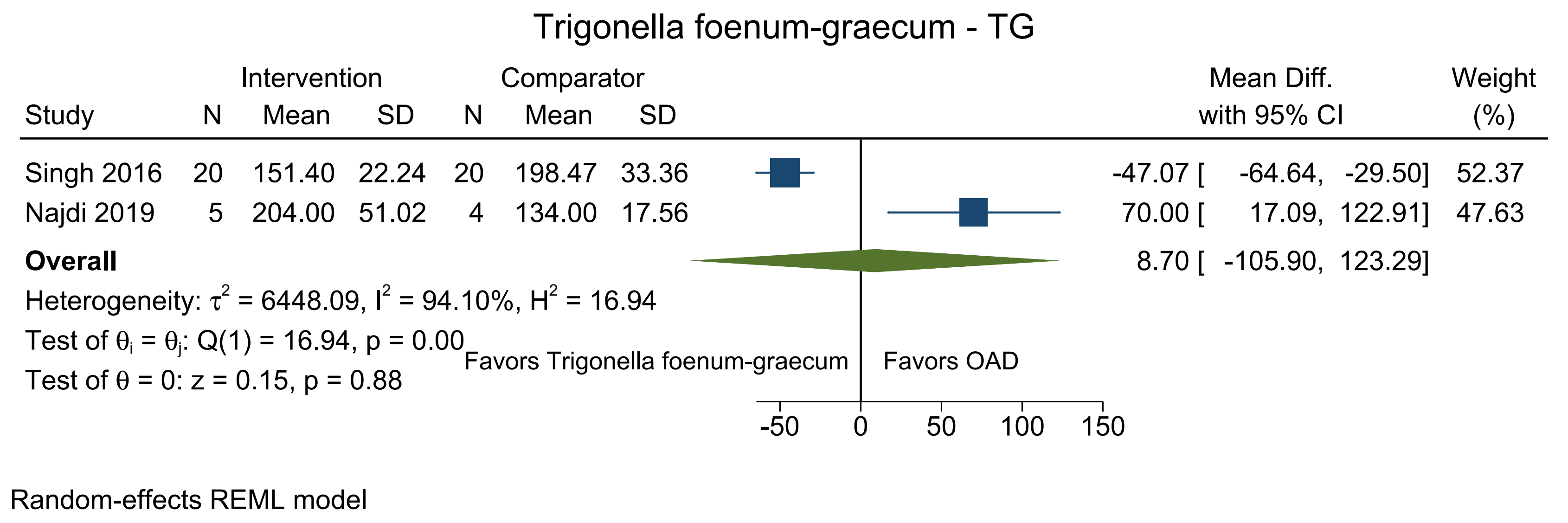
Trigonella foenum-graecum - TG
Intervention
Comparator
Study
N
Mean
SD
N
Mean
SD
Mean Diff.
with 95% CI
Weight
(%)
Singh 2016 20 151.40 22.24 20 198.47 33.36 -47.07 [-64.64, -29.50] 52.37
Najdi 2019 5 204.00 51.02 4 134.00 17.56 70.00 [17.09, 122.91] 47.63
Overall 8.70 [-105.90, 123.29]
Heterogeneity: τ² = 6448.09, I² = 94.10%, H² = 16.94
Test of θi = θj: Q(1) = 16.94, p = 0.00
Test of θ = 0: z = 0.15, p = 0.88
Favors Trigonella foenum-graecum
Favors OAD
-50
0
50
100
150
Random-effects REML model
